# SGLT2 inhibitors act from the extracellular surface of the cell membrane

**DOI:** 10.14814/phy2.12058

**Published:** 2014-06-27

**Authors:** Chiara Ghezzi, Bruce A. Hirayama, Edurne Gorraitz, Donald D. F. Loo, Yin Liang, Ernest M. Wright

**Affiliations:** 1 Department of Physiology, Geffen School of Medicine at UCLA, Los Angeles, California; 2 Janssen Research and Development, LLC, Spring House, Pennsylvania

**Keywords:** Inhibitors, SGLT2, type II diabetes

## Abstract

SGLT2 inhibitors are a new class of drugs that have been recently developed to treat type II diabetes. They lower glucose levels by inhibiting the renal Na^+^/glucose cotransporter SGLT2, thereby increasing the amount of glucose excreted in the urine. Pharmacodynamics studies have raised questions about how these inhibitors reach SGLT2 in the brush border membrane of the S1 and S2 segments of the renal proximal tubule: are these drugs filtered by the glomerulus and act extracellularly, or do they enter the cell and act intracellularly? To address this question we expressed hSGLT2 in HEK‐293T cells and determined the affinity of a specific hSGLT2 inhibitor, TA‐3404 (also known as JNJ‐30980924), from the extra‐ and intracellular side of the plasma membrane. Inhibition of SGLT2 activity (Na^+^/glucose currents) by TA‐3404 was determined using the whole‐cell patch clamp that allows controlling the composition of both the extracellular and intracellular solutions. We compared the results to those obtained using the nonselective SGLT inhibitor phlorizin, and to the effect of TA‐3404 on hSGLT1. Our results showed that TA‐3404 is a potent extracellular inhibitor of glucose inward SGLT2 transport (IC_50_ 2 nmol/L) but it was ineffective from the intracellular compartment at both low (5 mmol/L) and high (150 mmol/L) intracellular NaCl concentrations. We conclude that TA‐3404 only inhibits SGLT2 from the extracellular side of the plasma membrane, suggesting that it is filtered from the blood through the glomerulus and acts from within the tubule lumen.

## Abstract (precis)

An emerging class of new drugs to treat diabetes is the SGLT2 inhibitors which control blood glucose levels by excreting glucose into the urine. Questions have arisen about how these inhibitors reach SGLT2 in the brush border membrane of the S1 and S2 segments of the renal proximal tubule. Using patch‐clamp technique, we characterized the effect of a canagliflozin derivative (TA‐3404) on glucose‐induced currents and we showed that the inhibitor binds to SGLT2 from the extracellular side of the plasma membrane. This provides indirect evidence that SGLT2‐specific drugs act from the tubular lumen and not from the blood through the tubular epithelium.

## Introduction

Sodium‐coupled glucose transporters (or SGLTs) are a large class of proteins that mediate the thermodynamically coupled transport of sugars and Na^+^ across the plasma membrane of cells from a wide variety of tissues (for an extensive review see (Wright et al. 2011)). In the kidney, reabsorption of glucose is primarily mediated by two members of the SGLT family located on the luminal membrane of the proximal tubule (Wright et al. 2011). SGLT2, is found in the early portion of the renal proximal tubule (S1 and S2 segments) and normally accounts for the bulk of glucose reabsorption. SGLT1, is found in the more distal parts of the proximal tubule (S3 segment). Under physiological conditions, it has been estimated that SGLT2 is responsible for the absorption of ~80–90% of the filtered glucose load while the remaining ~10–20% of filtered glucose is taken up by the SGLT1 transporter (Wright et al. 2011; Abdul‐Ghani et al. 2012). There is, however, considerable SGLT1 reserve capacity to reabsorb glucose when SGLT2 activity is compromised (Rieg et al. 2013). The efficiency of SGLTs in conserving glucose is a disadvantage in patients with type II diabetes for whom it would be desirable to increase the amount of filtered glucose excreted in the urine.

The pharmaceutical industry has successfully introduced a new class of drugs, SGLT2‐specific inhibitors, to treat diabetes (for a review see (Abdul‐Ghani et al. 2012)). Two have been approved by the FDA, canagliflozin (Invokana^™^) (Nomura et al. 2010) and dapagliflozin (Farxiga^™^) (Han et al. 2008) and several others are either in advanced Phase III clinical trials or under review. In vivo these drugs partially block the reabsorption of glucose from the glomerular filtrate in the early proximal tubule. At therapeutic doses the result is the urinary excretion of up to 100 grams of glucose per day (Devineni et al. 2013; Scheen 2014) and a therapeutically relevant lowering of plasma glucose concentration without producing hypoglycemia.

In vitro these drugs in the extracellular media produce a specific, rapid, and reversible reduction of glucose transport by SGLT2. Pharmacokinetic studies in human and animal models have shown that once a day oral drug doses produce a sustained reversible glucosuria and that the major pathway for drug excretion is through the liver and bile and not the urine (Seman et al. 2013; Kasichayanula et al. 2014; Mamidi et al. 2014). This is perhaps uprising in that the inhibitors are expected to act on SGLT2 in S1 and S2 segments from the lumen of the proximal tubule and would pass to urine after saturation of SGLT2 binding sites. In contrast, the parent SGLT inhibitor phlorizin is mainly excreted into the urine after glomerular filtration and binding to SGLT1 and 2 in the apical membrane of the tubule (Silverman 1974). Phlorizin only inhibits SGLT1 from the external surface of the cell membrane (Eskandari et al. 2005). This discrepancy between the SGLT2 drugs and phlorizin has raised questions about their site of action: namely, are the specific SGLT2 inhibitors not filtered at the glomerular apparatus because of binding to plasma proteins; or do drugs act only from the basolateral (serosal) side by permeating the basolateral membrane and acting on the cytoplasmic surface of SGLT2 in the apical membrane?

**Figure 1.**
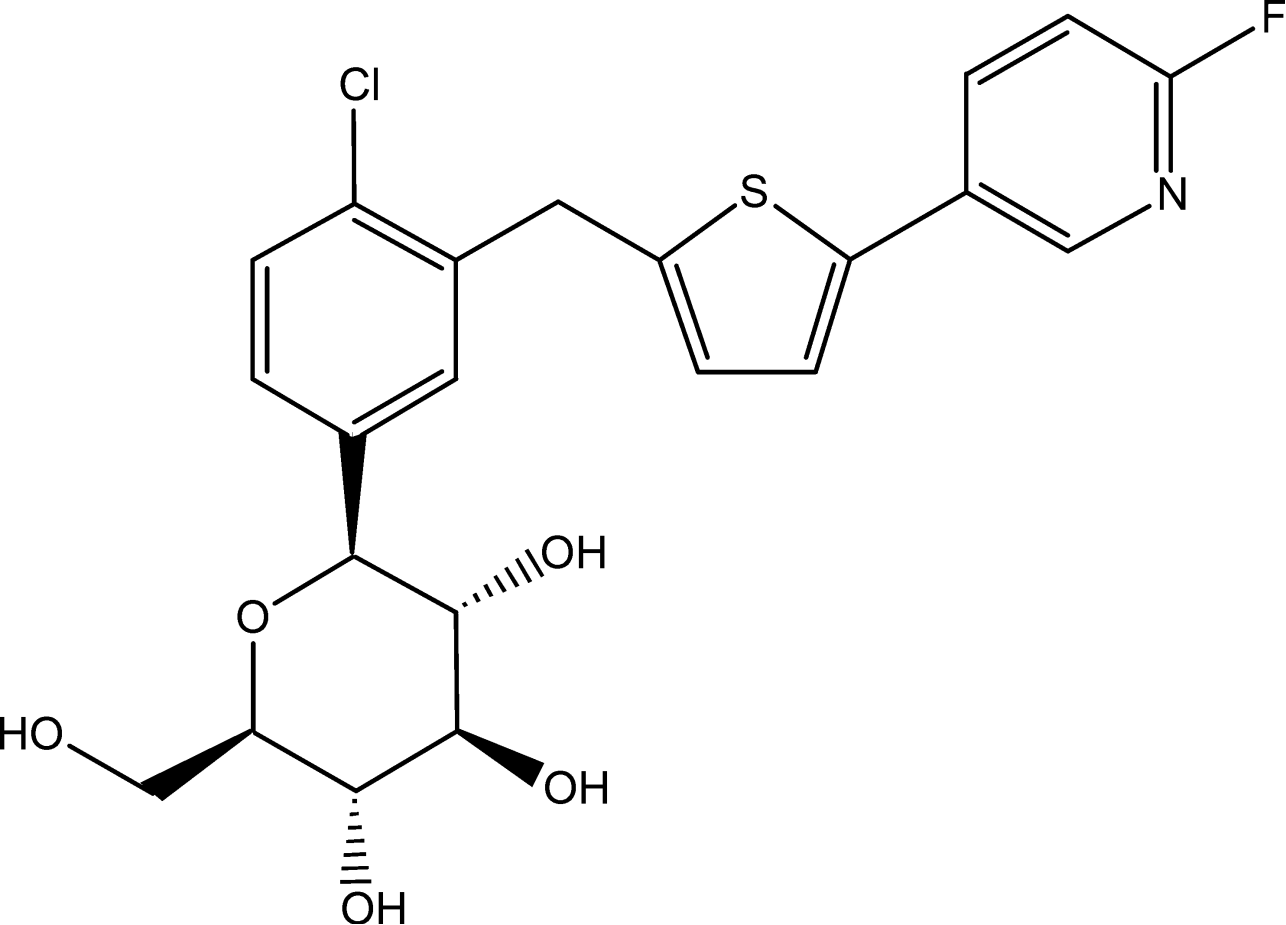
Chemical structure of TA‐3404. Chemical structure of JNJ‐30980924 (TA‐3404) or Chloro‐3‐(5‐(6‐fluoro‐3‐pyridyl)‐2‐thienylmethyl)‐1‐(*β*‐D‐glucopyranosyl) benzene.

In this study, we have directly tested if the specific SGLT2 inhibitor JNJ‐30980924/TA‐3404 (Koga et al. 2013) (Fig. 1) can inhibit hSGLT2 from the cytosolic surface using a whole‐cell patch‐clamp method.

## Methods

### Reagents and solutions

The standard extracellular solution (Na^+^ buffer) contained, in mmol/L, 150 NaCl, 1 CaCl_2_, 1 MgCl_2_, 10 HEPES/TRIS, pH 7.4. For Na^+^‐free solution (Choline buffer) NaCl was equimolarly replaced with cholineCl. For whole‐cell patch‐clamp experiments at 37°C, mannitol (100 mmol/L) was added to the extracellular solution to reduce noise and improve stability in the whole‐cell recording mode. The standard intracellular solution (pipette solution) contained, in mmol/L, 145 CsCl, 5 NaCl, 11 EGTA and 10 HEPES/TRIS, pH 7.4. High Na^+^ intracellular solution contained in mmol/L: 150 NaCl, 11 EGTA and 10 HEPES/TRIS, pH 7.4.

JNJ‐30980924/TA‐3404 (Fig. 1) (Janssen Research & Development LLC) stock solution was prepared in DMSO at a concentration of 100 mmol/L (Koga et al. 2013).

### Cell culture and transfection

The protocol that we used to grow and transfect the cells have been already described elsewhere (Hummel et al. 2011). In brief, human embryonic kidney 293T (HEK‐293T) cells were purchased from the American Type Culture Collection (Manassas, VA). Cells were grown in vented 25‐cm^2^ polystyrene flasks, in Dulbecco's modified Eagle's medium (DMEM, CELLGRO, Manassa, VA) supplemented with 10% fetal calf serum (Valley Biomedical Products, Winchester, VA) and 1% penicillin‐streptomycin (Invitrogen, Carlsbad, CA). Cells were maintained in incubator at 37°C in humidified atmosphere of 5% CO_2_ and 95% air. Cells were passaged (1:10) every 5 days, and before transfection were seeded in six‐well plates or poly‐L‐lysine‐coated 24‐well plates. Cells were grown till 50–70% confluence and then transfected with 1 *μ*g of the vector containing the hSGLT2 or hSGLT1 coding region (IRES‐hSGLT2 or IRES‐hSGLT1) using the Effectene kit (Qiagen, Germantown, MD).

### [^14^C]‐*α*‐Methyl‐D‐Glucopyranoside (*α*‐MDG) uptake

Sugar uptake was measured in HEK‐293T cells expressing hSGLT1 or hSGLT2 as described (Hummel et al. 2011). In brief, cells were incubated for 30 min in 50 *μ*mol/L [^14^C]‐*α*‐Methyl‐D‐glucopyranoside (GE HealthCare Life Sciences, Piscataway, NJ), a specific substrate for SGLTs, and washed in cold Choline buffer three times. The cells were solubilized and a sample was assayed using scintillation counting. Uptake was normalized to total cellular protein. For each tested condition, the sample size was *n* = 3–4 wells and each experiment was repeated at least twice. Uptakes were measured in the presence and absence of 100 *μ*mol/L phlorizin or the indicated concentration of TA‐3404.

### Whole‐cell patch‐clamp recording

The procedure for whole‐cell patch‐clamp experiments has been explained elsewhere (Hummel et al. 2011). In brief, 2 days after transfection cells were plated on 12‐mm poly‐L‐lysine‐coated glass coverslips and selected on the basis of fluorescence intensity using a Nikon diaphot epifluorescence microscope (Nikon, Tokyo, Japan). During the experiment cells were kept at the holding potential *V*_*h*_ = −60 mV. For hSGLT2 experiments, solutions were heated at 37°C via an in‐line solution heater (TC‐324B Warner Instrument Corporation, CT), whereas for hSGLT1, experiments were carried out at 22°C. Currents were filtered at 2 kHz and digitized at 1 kHz.

Glucose currents (*I*_Glu_) were obtained by subtracting the baseline current recorded in Na^+^ buffer to the total current measured in Na^+^ buffer + glucose: 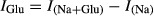


To measure the effect of the inhibitor from the cytoplasmic side, we add phlorizin or TA‐3404 to the pipette intracellular solution and we compared the inward glucose‐induced current measured in presence or absence of the inhibitor.

### Inhibitor kinetic

To study TA‐3404 interaction with the SGLTs we used the same approach described by Hummel et al. (2011) to measure dapagliflozin and phlorizin interaction with hSGLT1 and hSGLT2. The inhibition constant (IC_50_) was determined by measuring the sugar‐dependent steady‐state current or radiotracer uptake as a function of external inhibitor concentration. In these experiments, the glucose concentration was close to the *K*_0.5_ of the sugar so the concentration of the inhibitor producing 50% inhibition (IC_50_) is twice the *K*_*i*_ (Loo et al. 2008; Hummel et al. 2012).

Inhibitor off rates (*k*_off_) were determined by recording the time course of recovery of the D‐glucose‐coupled current after rapid removal of the inhibitor, assuming pseudo‐first‐order binding and dissociation kinetics (Hummel et al. 2011): 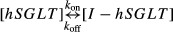


where I is the inhibitor tested, *k*_on_ is the association rate, and *k*_off_ is the dissociation rate.

The D‐glucose‐coupled current recovery follows an exponential time course with time constant 1/*k*_off_ and half‐time for recovery (*t*_1/2,off_) related to the off rate: 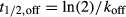


The ON rate (*k*_on_) is calculated from the empirically measured *K*_*i*_ values and *k*_off_ values: 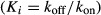


### Data analysis

Statistical analysis was made using GraphPad Prism version 4 for Windows, GraphPad Software (San Diego, CA). In the graphs, data are shown as mean ± SE.

## Results

### TA‐3404 is a selective hSGLT2 inhibitor

**Figure 2.**
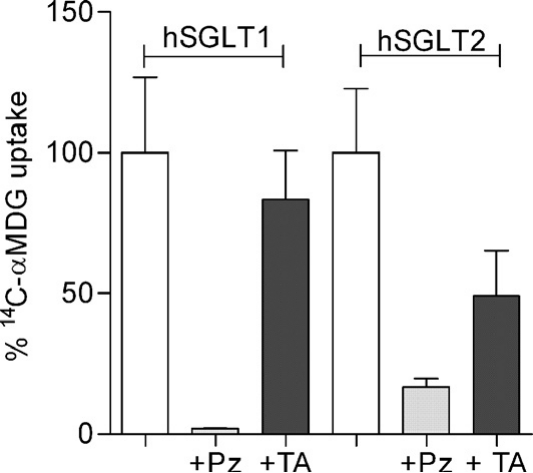
Inhibitors effect on [^14^C]‐*α*MDG uptake mediated by hSGLT1 and hSGLT2. [^14^C]‐*α*MDG uptake was measured in HEK‐293T cells expressing hSGLT1 or hSGLT2 in control conditions (*white bars*), in presence of 100 *μ*mol/L phlorizin (Pz, *light gray bars*) and 300 nmol/L (hSGLT1) or 2 nmol/L (hSGLT2) TA‐3404 (TA, *dark gray bars*). Uptake was measured at 37°C and expressed as quantity of tracer (pmol) per minute per *μ*g protein. Bars are means ± SE, *n* = 4 wells.

Previous studies (Koga et al. 2013) have shown that, in CHOK1 cells overexpressing hSGLT1 or hSGLT2, TA‐3404 is a potent, selective inhibitor for hSGLT2. TA‐3404 inhibited Na^+^‐dependent ^14^C‐*α*‐methylglucoside (*α*‐MDG) uptake with an IC_50_ of 2 nmol/L against SGLT2 and 750 nmol/L against SGLT1 (~350‐fold selectivity for SGLT2 vs. SGLT1). Hereby, we decided to confirm these results investigating the effect of TA‐3404 on hSGLT1 or hSGLT2 activity in HEK‐293T cells. Uptakes of 50 *μ*mol/L *α*‐MDG into the transfected cells were measured in the presence and absence of inhibitors (Fig. 2). 100 *μ*mol/L phlorizin (light gray bars in Fig 2) reduced the sugar uptakes by hSGLT1 and hSGLT2 to the level observed in untransfected cells. In contrast, 300 nmol/L TA‐3404 (dark bars in Fig. 2) inhibited hSGLT1 uptake by only 10% and 2 nmol/L TA‐3404 inhibited hSGLT2 sugar uptake by 50%.

**Figure 3.**
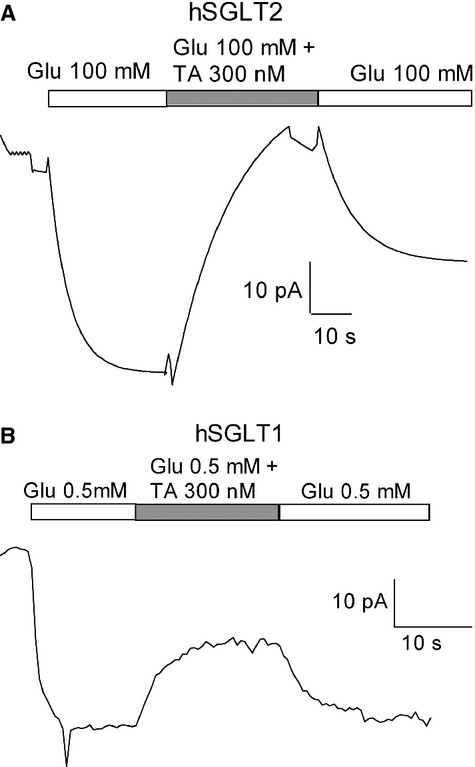
TA‐3404 effect on glucose‐induced currents. (A) Current recording obtained from cells expressing hSGLT2 at a *V*_*h*_ = −60 mV and at 37°C. Glucose‐dependent current was induced by adding 100 mmol/L glucose to the extracellular solution. The time course of inhibition of glucose‐induced current was monitored upon application of TA‐3404 (TA) and upon removal of the inhibitor, the time course of current recovery. (B) Current recording obtained from cells expressing hSGLT1 at a *V*_*h*_ = −60 mV and at 22°C. Glucose‐dependent current was induced by adding 0.5 mmol/L glucose to the extracellular solution. The time course of inhibition of glucose‐induced current was monitored upon application of TA‐3404 (TA) and upon removal of the inhibitor, the time course of current recovery.

Similar studies were carried out using the whole‐cell patch‐clamp method on HEK‐293T cells expressing hSGLT1 and hSGLT2 (Hummel et al. 2011, 2012; Ghezzi and Wright 2012). The advantage of this technique is that it allows us to measure the effect of the inhibitor on glucose‐induced currents at a constant membrane potential (−60 mV). Figure 3A and B shows experiments recording the effect of TA‐3404 on hSGLT2 and hSGLT1 currents, respectively. In a cell expressing SGLT2, addition of 100 mmol/L glucose to the superfusate (Na^+^ buffer) generated an inward current of 37 pA that was completely inhibited by the addition of 300 nmol/L TA‐3404 (Fig. 3A). Figure 3B shows the time course of the inhibition of the Na^+^/glucose current recorded on HEK‐293T cells transfected with hSGLT1. Superfusion of the cell with 0.5 mmol/L glucose produced an inward current of 28 pA. Addition of 300 nmol/L TA‐3404 reduced the current by 35%, to a new steady state of 20 pA.

### Determination of TA‐3404 *K*_i_, *k*_on_, and *k*_off_ for hSGLT1 and hSGLT2

The experimental approach to calculate *K*_i_, *k*_on_, and *k*_off_ is the same as previously used to calculate the binding properties of phlorizin and dapagliflozin to both hSGLT1 and hSGLT2 (Hummel et al. 2011). To calculate the dissociation constant (*k*_off_) we measured the recovery time of SGLT glucose‐induced current when the inhibitor is rapidly removed from the extracellular solution.

For hSGLT2 (Fig. 3A) removing the inhibitor from the perfusate resulted in recovery of only ~50% of the hSGLT2 current with a half‐time of 17 sec (17 ± 0.5 (*n* = 3) seconds). In three experiments, there was no further recovery of the hSGLT2 current over 5 min. This is contrary to the results where phlorizin inhibition was 100% reversible, but the time course for the incomplete reversal was similar to phlorizin (17 vs. 24 sec (see (Hummel et al. 2011)).

For hSGLT1 (Fig. 3B) washing with extracellular buffer free of TA‐3404 reversed the hSGLT1 current with a half‐time of 5.4 ± 0.4 (*n* = 5) seconds, comparable to the half‐time for phlorizin on hSGLT1 (Hummel et al. 2011). For both inhibitors this corresponds to a *k*_off_ of 0.2 per sec.

**Figure 4.**
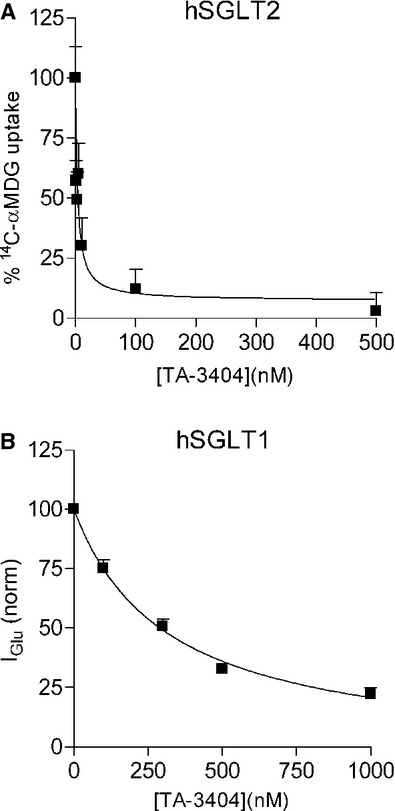
Inhibition of hSGLT1 and hSGLT2 by extracellular TA‐3404. (A) [^14^C]‐*α*MDG (50 *μ*mol/L) uptake was measured in cells expressing hSGLT2 in control condition and in presence of 1, 3, 5, 10, 100, and 500 nmol/L TA‐3404. Bars are means ± SE, *n* = 4 wells. (B) Current induced by 0.5 mmol/L glucose was recorded from cells expressing hSGLT1 in control condition and in presence of 100, 300, 500, and 1000 nmol/L TA‐3404. Bars are means ± SE, *n* = 4 wells.

We calculate the inhibition constant (*K*_*i*_) by measuring the effect of increasing concentrations of TA‐3404 on radiotracer uptake (for hSGLT2) or glucose‐induced current (for hSGLT1). We decided to use two different approaches due to the fact that hSGLT2 has low expression, yielding small glucose‐induced currents. For radiotracer uptake experiments, tracer entry in the cell is measured over the period of 30 min leading to a higher sensitivity of the method. For hSGLT2 (Fig. 4A) we measured the uptake of 50 *μ*mol/L [^14^C]‐*α*MDG in presence of 1, 3, 5, 10, 100, 500 nmol/L TA‐3404. From these data, we estimated an IC_50_ of 3.9 ± 2.2 nmol/L that correspond to a *K*_*i*_ of ~2 nmol/L. For hSGLT1 (Fig. 4B) 50% inhibition of the 0.5 mmol/L glucose current was estimated to occur at 300 ± 61 nmol/L that corresponds to a *K*_*i*_ of 150 nmol/L.

From these results we were able to calculate the *k*_on_ (*k*_on_ = *K*_*i*_ × *k*_off_): for hSGLT1 we computed a *k*_on_ of 25 × 10^6 ^mol/L per sec, whereas for hSGLT2 of 1.4 × 10^6^ mol/L per sec.

### TA‐3404 acts only from the extracellular side

Our results showed that TA‐3404 is a potent extracellular inhibitor of glucose transport by hSGLT2 in HEK‐293T cells with an inhibition constant (*K*_*i*_ ~2 nmol/L) that is similar to the one measured for phlorizin and drugs such as dapagiflozin (Hummel et al. 2011, 2012). As expected, TA‐3404 inhibited glucose transport mediated by hSGLT1 with a higher *K*_i_, 150 nmol/L indicating that at therapeutic concentrations TA‐3404 is a more potent hSGLT2 inhibitor.

**Figure 5.**
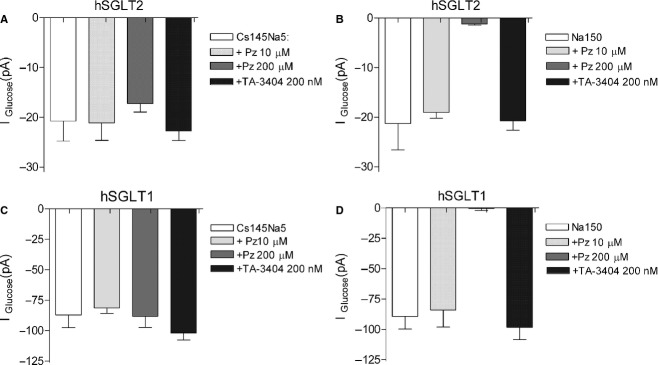
Sidedness of TA‐3404 interaction for hSGLT2 and hSGLT1. Inward glucose currents were measured in cells expressing hSGLT2 (A and B) and hSGLT1 (C and D) as a function of the intracellular inhibitors (phlorizin and TA‐3404) in 5 or 150 mmol/L NaCl). All cells were incubated in standard 150 mmol/L NaCl buffer at 37°C in the presence and absence of glucose (100 mmol/L for hSGLT2, and 10 mmol/L for hSGLT1) at a holding potential of −60 mV. (A) hSGLT2 inward glucose currents measured in the presence of 5 mmol/L NaCl intracellular buffers before and after the addition of 10 *μ*mol/L phlorizin, 200 *μ*mol/L phlorizin or 200 nmol/L TA‐3404. Bars are means ± SE, *n* = 4 cells. (B) hSGLT2 inward glucose currents measured in the presence of 150 mmol/L NaCl intracellular buffers before and after adding the addition of 10 *μ*mol/L phlorizin, 200 *μ*mol/L phlorizin or 200 nmol/L TA‐3404. Bars are means ± SE, *n* = 4. (C) hSGLT1 inward glucose currents measured in the presence of 5 mmol/L NaCl intracellular buffers before and after the addition of 10 *μ*mol/L phlorizin, 200 *μ*mol/L phlorizin or 200 nmol/L TA‐3404. Bars are means ± SE, *n* = 4 cells. (D) hSGLT1 inward glucose currents measured in the presence of 150 mmol/L NaCl intracellular buffers before and after adding the addition of 10 *μ*mol/L phlorizin, 200 *μ*mol/L phlorizin or 200 nmol/L TA‐3404. Bars are means ± SE, *n* = 4.

The sidedness of TA‐3404 interaction with hSGLT2 and hSGLT1 was determined by recording the inhibition of inward Na^+^/glucose currents when the drug was delivered to the intracellular compartment using the whole‐cell patch‐clamp method. Phlorizin was used as a control. Our strategy was to measure the inward Na^+^/glucose current at −60 mV as a function of the intracellular TA‐3404. The intracellular NaCl concentration was maintained at either 5 or 150 mmol/L using CsCl to replace NaCl (Fig. 5A and B).

Figure 5A summarizes experiments on the intracellular inhibition of inward hSGLT2 Na^+^/glucose currents by TA‐3404 and phlorizin. In control condition, with an intracellular Na^+^ concentration of 5 mmol/L, we measured a mean current of ~20 pA. Addition of 10–200 *μ*mol/L phlorizin or 200 nmol/L TA‐3404 to the intracellular solution did not inhibit the current.

We repeated the experiments on hSGLT2 cells, now using a high Na^+^ intracellular solution (150 mmol/L) (Fig. 5B). In this case, there was no significant inhibition when 10 *μ*mol/L phlorizin or 200 nmol/L TA‐3404 were added to the intracellular solution but we observe a dramatic decrease (85%) in the glucose‐induced current with the addition of 200 *μ*mol/L phlorizin.

Figure 5C presents a summary for hSGLT1. With 5 mmol/L NaCl in the pipette the inward Na^+^/glucose current was ~85 pA. There was no effect when 10 and 200 *μ*mol/L phlorizin or 200 nmol/L TA‐3404 were added to the intracellular solution. Figure 5D shows the effect of inhibitors when the pipette was loaded with high Na^+^ solution (150 mmol/L). As observed for hSGLT2, we did not detect any significant effect on the glucose‐induced current by 10 *μ*mol/L phlorizin or 200 nmol/L TA‐3404, but there was a dramatic decrease in current when 200 *μ*mol/L phlorizin was present in the intracellular solution.

## Discussion

Recently, many hSGLT2 inhibitors have been developed with the aim of reducing the plasma glucose levels in patients with type II diabetes (Vallon 2010; Abdul‐Ghani et al. 2012). In vitro these inhibitors (e.g., dapagliflozin) block hSGLT2‐mediated glucose transport capability with high affinity, *K*_*i*_ 6 nmol/L, but are relatively poor inhibitors of hSGLT1, *K*_*i*_ 390 nmol/L (Hummel et al. 2011). Although clinical trials (Komoroski et al. 2009; Devineni et al. 2013) have shown that these drugs are effective in reducing plasma glucose concentration, little is known about the mechanism of action in vivo. In particular, questions have arisen about how these inhibitors reach SGLT2 in the brush border membrane of the S1 and S2 segments of the renal proximal tubule: are these drugs filtered by the glomerulus and act from within the tubule, or do they act intracellularly, gaining access to SGLT2 from the blood side of the cell?

To answer this question we expressed hSGLT1 and hSGLT2 in HEK‐293T cells and tested the effect of two different inhibitors, phlorizin and TA‐3404, when they were applied to the extracellular and intracellular compartments.

TA‐3404 is a derivative of canagliflozin (Koga et al. 2013) with an IC_50_ for hSGLT2 of 2 nmol/L. Canagliflozin has been demonstrated to be a highly potent and selective inhibitor of hSGLT2. The IC_50_ calculated for both hSGLT1 and hSGLT2 indicate that the drug has a 414‐fold selectivity for hSGLT2 over hSGLT1 (Nomura et al. 2010). In order to improve the specificity and potency of Canagliflozin, different derivates have been developed and studied (Koga et al. 2013). In particular among C‐glucosides conjugated to heteroaryl thiophenes, the 6‐fluoro‐3‐pyridylthiophene derivate, TA‐3404, showed favorable pharmacokinetic profiles and an increased urine glucose excretion.

The aim of this study was to understand how SGLT2 inhibitors interact with SGLT cotransporter proteins to inhibit renal glucose reabsorption by using biochemical and biophysical techniques. Also, to determine the cellular site of hSGLT2 activity of TA‐3404, luminal versus cytosol, by determining the exposure response relationships in vitro using the whole‐cell patch‐clamp method to deliver the molecule inside the cell.

Our study confirmed that TA‐3404 is a high affinity, selective inhibitor of glucose transport by human SGLT2 expressed in cultured cells (Koga et al. 2013). The affinity of hSGLT2 for TA‐3404 was about 75‐fold higher than for hSGLT1 expressed in HEK‐293T (Fig. 2). These results are comparable to those obtained for canagliflozin using similar in vitro assays (Nomura et al. 2010).

Examination of the On and Off rates for inhibition of glucose (Fig. 3A and B) transport revealed that: (1) TA‐3404 and phlorizin interactions with hSGLT1 are very similar. The *K*_*i*_ for TA‐3404 and phlorizin was 150 nmol/L and the On and Off rates were 1.4 × 10^6^ mol/L per sec and 0.2 per sec; and (2) TA‐3404 binding to hSGLT2 probably involves two components: a reversible component with an Off rate close to that for phlorizin, 0.3 per sec, and a component with an extremely slow dissociation constant. This interesting property of TA‐3404 binding to hSGLT2 warrants further investigation. It should be noted that dapagliflozin binding to hSGLT2 exhibits a very slow Off rate (Hummel et al. 2011), and this in part accounts for both the high affinity of this inhibitor and the pharmacokinetics in vivo.

The most novel finding is that, under physiological conditions, TA‐3404 in the cytoplasm of HEK293T cells does not inhibit Na^+^/glucose currents generated by hSGLT2 or hSGLT1 (Fig. 5) indicating that the intracellular affinity of hSGLT2 for TA‐3404 is at least 100 lower than the extracellular affinity, in agreement with previous studies (Eskandari et al. 2005) showing that phlorizin is a poor intracellular inhibitor of SGLT1. When applied from the intracellular compartment, the inhibition constant, *K*_*i*_, for SGLT1 increased from ~10 *μ*mol/L (value measured when phlorizin is applied from outside (Hirayama et al. 1996; Umbach et al. 1990)) to >1 mmol/L.

Our results indicate that, like phlorizin, TA‐3404 inhibits SGLT2 by acting from the lumen of the proximal tubule. Earlier studies have shown that phlorizin is freely filtered and completely blocks glucose reabsorption (Chasis et al. 1933; Silverman 1974). All of the specific SGLT2 inhibitors described so far have similar pharmacokinetic and pharmacodynamic profiles, that is, after a single clinical oral dose they reach a maximum plasma concentration in 1–2 h and have a plasma half‐life of 10–15 h (Komoroski et al. 2009; Obermeier et al. 2010; Devineni et al. 2013; Seman et al. 2013; Scheen 2014). Even though 90% of the drugs are bound to plasma proteins, we have estimated that the free plasma concentration after 24 h is significantly higher than the SGLT2 inhibitor constant, for example, for dapagliflozin the free plasma concentration is 25 nmol/L which is fivefold higher than the *K*_*i*_ (5 nmol/L) (Hummel et al. 2012). We propose that there is sufficient drug in the glomerular filtrate to bind and inhibit SGLT2 in the S1 and S2 segments of the proximal tubule. At maximum oral drug doses only 55% of the filtered glucose load is blocked from reabsorption due to the reserve capacity of SGLT1 in the S3 segment (Rieg et al. 2013).

As little as 2% of the unmodified drug is excreted into the urine and this suggests that much of the filtered load of drug is reabsorbed beyond the proximal tubule. We suggest that the variation in the amount of free drugs lost to the urine (2–18%) is due to the selectivity and kinetics of a transporter responsible for reabsorption. The bulk of the drug in circulation is taken up by the liver and excreted as free drug and/or metabolites in the bile.

In summary, our results provide indirect evident that SGLT2 inhibitors block the reabsorption of glucose in the early proximal tubule from the glomerular filtrate and that the drugs are reabsorbed further down the tubule. Additional experiments are required to provide direct tests our hypothesis.

## Acknowledgment

We thank Erika Patino for preliminary experiments on the effect of TA‐3404 on hSGLT1 in *Xenopus laevis* oocytes.

## Conflict of interest

None declared.
